# Mosaic Loss of Chromosome Y and Plasma Aβ42/p‐tau181 Ratio as Biomarkers for Alzheimer's Disease in the Midwestern Amish

**DOI:** 10.1002/alz70856_106620

**Published:** 2026-01-08

**Authors:** Yeunjoo E. Song, Ping Wang, Renee A. Laux, Sarada L. Fuzzell, Sherri D. Hochstetler, Kristy L. Miskimen, Audrey Lynn, Weihuan Wang, Yining Liu, Noel C. Moore, Alex V. Gulyayev, Daniel A. Dorfsman, Laura J. Caywood, Jason E. Clouse, Sharlene D. Herington, Michael B. Prough, Susan H. Slifer, Larry D Adams, Patrice G Whitehead, Jeffery M Vance, Michael L Cuccaro, Paula K. Ogrocki, Alan J. Lerner, Margaret Pericak‐Vance, William K. Scott, William S Bush, Jonathan L Haines

**Affiliations:** ^1^ Department of Population and Quantitative Health Sciences, Case Western Reserve University School of Medicine, Cleveland, OH, USA; ^2^ Cleveland Institute for Computational Biology, Case Western Reserve University, Cleveland, OH, USA; ^3^ John P. Hussman Institute for Human Genomics, University of Miami Miller School of Medicine, Miami, FL, USA; ^4^ Dr. John T. Macdonald Foundation Department of Human Genetics, University of Miami Miller School of Medicine, Miami, FL, USA; ^5^ University of Miami Miller School of Medicine, Miami, FL, USA; ^6^ Department of Neurology, Case Western Reserve University School of Medicine, Cleveland, OH, USA; ^7^ Department of Neurology, University Hospitals Cleveland Medical Center, Cleveland, OH, USA

## Abstract

**Background:**

In aging men, mosaic loss of chromosome Y (mLOY) is a possible biomarker for increased risk of disease, including Alzheimer disease (AD). We previously reported mLOY increased with age and carriers of mLOY had an increased risk of AD. We also found plasma Aβ42/p‐tau181 ratio (APR) was significantly lower among AD Amish individuals compared to cognitively‐unimpaired (CU) individuals. We now examine how these two biomarkers interplay with cognitive status in Amish males.

**Method:**

mLOY was determined using the Mosaic Chromosomal Alterations(MoChA) pipeline. Extensive QC was done for both mLOY and plasma biomarker measures. Consensus review of medical history and neuropsychological testing categorized individuals into AD, Mild‐cognitive‐impairment (MCI) or Cognitive‐impairment‐not‐AD (CINAD) or CU. The cognitively‐impaired (CI) group combined AD, MCI and CINAD. We compared 1) CI to CU and 2) AD to CU. Correlation between APR and mLOY were estimated accounting for relatedness. Receiver operating characteristic analysis was performed to evaluate the discriminatory ability of the two biomarkers compared to the baseline model with age and presence/absence of APOE ε4 alleles. *p*‐value <0.05 was noted as statistically significant.

**Result:**

249 males (mean age=82.89±5.57) had measurements for both biomarkers. Of these, a subset had consensus diagnoses AD (*n* = 30; mean age=85.63±5.31), CINAD, MCI or CU (*n* = 105; mean age=82.01±5.36). The mean age of 82 CI individuals was 84.34±4.73. mLOY was observed in 20.1% of CU vs. 29.3% of CI (*p*‐value=0.23). APR was significantly negatively correlated with CI and AD as expected, was positively correlated with mLOY but not significantly and remained positively correlated when stratified, with the stronger correlation in AD. For AD, the area under the curve (AUC) improved from 0.70 to 0.82 with the inclusion of APR, with mLOY showing little effect. For CI, AUC improved from 0.63 to 0.68 by including mLOY, with no independent effect of APR. When including both in the model, AUC improves from 0.63 to 0.69.

**Conclusion:**

We observed two promising biomarkers of AD, mLOY and APR, both contribute discriminating AD and CI but differently. This and the stringer correlation in AD may indicate they are more specific to AD than non‐specific CI.

